# Individual variability of vascularization of the anterior papillary muscle within the right ventricle of human heart

**DOI:** 10.1371/journal.pone.0205786

**Published:** 2018-10-15

**Authors:** Miłosz Andrzej Zajączkowski, Andrej Gajić, Agata Kaczyńska, Stanisław Zajączkowski, Jarosław Kobiela, Rafał Kamiński, Adam Kosiński

**Affiliations:** 1 Department of Clinical Anatomy, Medical University of Gdańsk, Gdańsk, Poland; 2 Department for Pathology, Faculty for Veterinary Medicine, University of Sarajevo, Sarajevo, Bosnia and Herzegovina; 3 Department of Physiology, Medical University of Gdańsk, Gdańsk, Poland; 4 Department of General, Endocrine and Transplant Surgery, Medical University of Gdańsk, Gdańsk, Poland; Universidad Francisco de Vitoria, SPAIN

## Abstract

**Background:**

To date there is scarce published evidence reporting the dual blood supply reaching anterior papillary muscle (APM), which descends from both major coronary arteries. Such a vascular configuration can prevent the dysfunction of right ventricular entire valvular system in case of the occlusion of proximal part of either right coronary artery (RCA) or left coronary artery (LCA). The aim of our study was to determine the vascular pattern of APM blood supply which originates from two main coronary arteries, in the context of the APM and septomarginal trabecula (SMT) topography.

**Methods:**

The study was carried out using tissue obtained from 36 human hearts. The material was divided into four morphological types of SMT/APM arrangement. Vascularization and blood supply pattern of papillary muscle was investigated following the analysis of multiple tissue cross sections. The origin of APM arterial supply was traced back to both main coronary arteries. Cross-sectional area of the arteries was estimated at the base of APM and compared within mixed male-female population, aged 18–76.

**Results:**

We noted that as much as 78% of entire APM material had a blood supply vasculature originating from both LCA and RCA branches. In contrast, 22% of cases APM was supplied by a single coronary artery, while in each case it proved to be LCA. We have never found APM arterial supply provided exclusively by RCA. In case of double AMP blood supply an average of total cross-section area of the arteries branching from LCA, was noted to be in excess of two and a half times bigger in type III and more than two times bigger in type IV, as compared with the arteries originating from RCA.

**Conclusions:**

Our research confirm the possibility of double blood supply which vascularizes APM, but the finding does not necessarily apply in all cases. However, APM seems to be predominantly vascularized by arteries deriving from LCA, regardless of their morphological type.

## Introduction

The literature to date provides little evidence in relation to vascular system of anterior papillary muscle (APM) of human heart right ventricle (RV). Most studies on papillary muscles concentrate on the left ventricle while few address any aspects of papillary muscles in RV. Scarce studies report APM being vascularised by both major coronary arteries, whose branches form anastomoses. Such vascular configuration could prevent dysfunction of the entire valvular apparatus of RV, for example in case of occlusion of the proximal part of either right coronary artery (RCA) or left coronary artery (LCA). RV seems to have a kind of protection against ischemia [1]. Isolated right ventricular infarction (RVI) is responsible for only as much as 2% of cases of death caused by coronary heart diseases [2,3]. Researchers propose varying explanations. There are reports suggesting that blood nourishing RV may be delivered by a collateral circulation from left coronary artery (LCA). Besides, a lower mass of RV may be a factor in its lower oxygen demand that of the left ventricle (LV). Moreover, unlike in LV, RV may have a mechanism enabling both systolic and diastolic arterial circulation [4,5]. In most cases, the main reason of a RVI is occlusion of the right coronary branch or acute marginal branch of the right coronary artery [3,6,7].

Our previous analyses point out the particular importance of the relationship between APM arterial supply configuration and morphological type of the connection between this muscle and the septomarginal trabecula (SMT) [8]. The arrangement of blood vessels seems to be strictly determined by the reciprocal topography of these two structures. This observation may prove to be of clinical significance. Ischemia of papillary muscles often results in their dysfunction and may contribute to the ischemic tricuspid valve regurgitation [9]. Furthermore, ischemia can result in rupture of the papillary muscle, which may necessitate urgent surgical intervention [10,11].

Until today, heart morphology research remains indispensable in the context of dynamic development of cardiologic procedures, including interventional cardiology and cardiac surgery. Detailed knowledge of construction, especially vascularization patterns, of individual heart structures is essential for better understanding of pathological variations which may occur within its constitution or beyond. Data gathered from such studies may be of key importance in the process to develop procedures where the heart structure is preserved and potential complications–prevented.

Many cases of valvular insufficiency have been described as a result of papillary muscle rupture [9,12–14]. Heart murmurs of unknown origin, detected during heart auscultation, can also be a consequence of APM ischemia. Murmurs are caused by an abnormal movements of one of the leaflets during heart contraction. Voci et al. [15] postulated, that myocardial infarction can be responsible for APM dysfunction, providing that the blood supply comes exclusively from one coronary artery which may also result in heart murmurs.

Addressing this question, which has obvious clinical implications, we aim to determine the vascular pattern of blood supply of APM from the two main coronary arteries taking into account the morphological type of APM/SMT connection.

## Materials and methods

**Fig 1:**
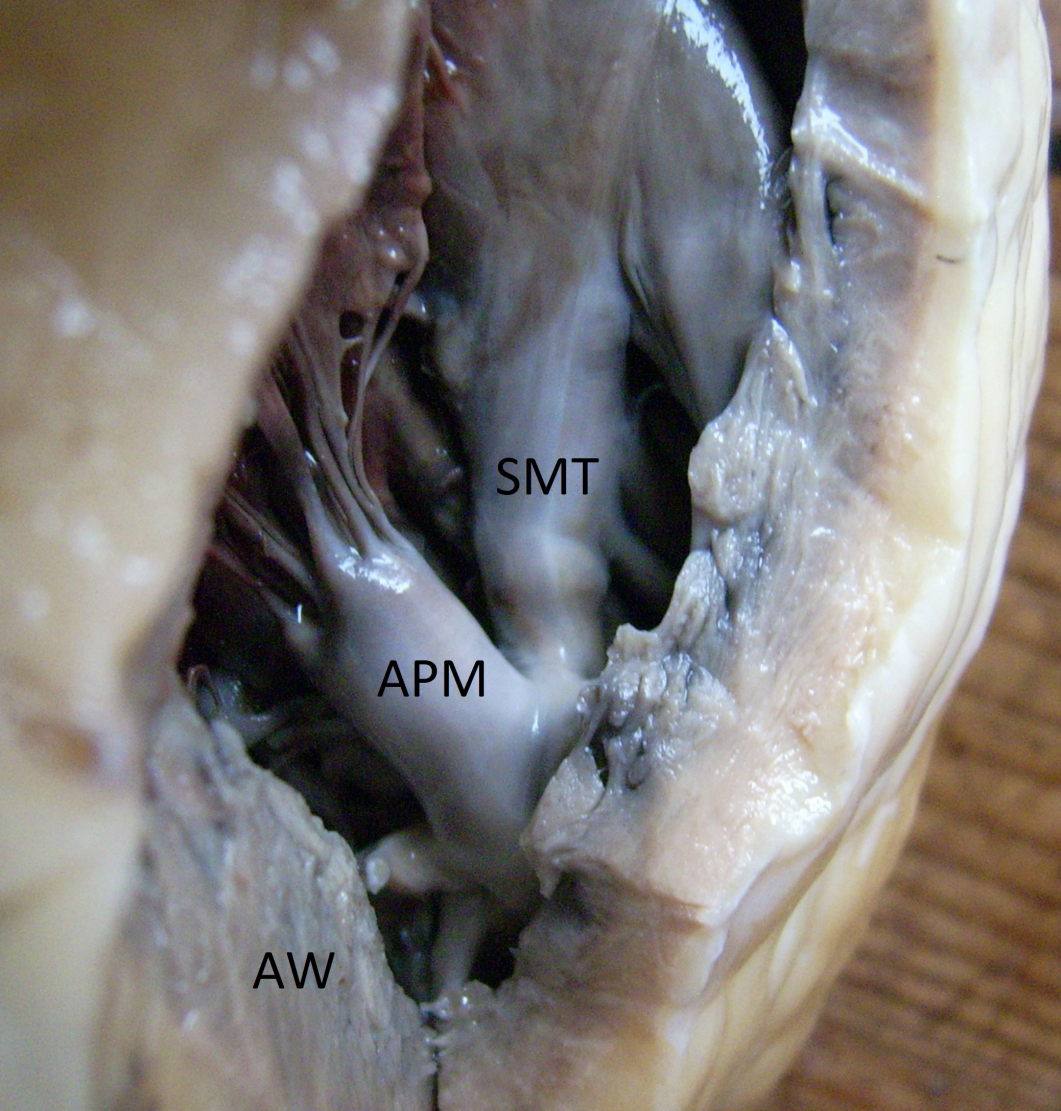
Interior view of the human heart; configuration type III: Permanent fusion of SMT within the APM. SMT–septomarginal trabecula, APM–anterior papillary muscle, AW–anterior wall of the right ventricle of the heart.

The protocol was approved by the Independent Bioethics Commission for Research of the Medical University of Gdańsk (consent no. 74/2012 dated 28 February 2012). The study was carried out using tissue from 36 human hearts derived from repository of the Department of Clinical Anatomy of Medical University of Gdańsk, Poland. Hearts were obtained from corpses of persons without a history of cardiovascular disease. The cause of death in each case was either an accident or a suicide. All hearts were instantly fixed in a solution of formalin and ethanol. The macroscopically seemingly healthy hearts, with no apparent lesions or developmental defects, were obtained from adults (18–76 years old) of both sexes. The material was divided into four morphological types, according to the classification developed by Kosinski [16]. The proposed four types (typology) refer to structural relation between SMT and APM and are characterised as follows: type I: SMT forms a relatively uniform, continuous structure not connected directly with APM; type II: SMT is in continuous or firm contact with APM; type III: permanent fusion of SMT with the body of APM (Fig 1); type IV: SMT is structurally divided in two by APM. In order to obtain an appropriate part of tissue, we attempted to excise a fragment, comprising a lower pole of supraventricular crest, SMT with APM and a portion of the anterior wall of right ventricle (AW). The specimens were embedded in paraffin and subsequently sectioned into 8-micrometer slices, using a microtome knife. The cuts were directed from the supraventricular crest through SMT and APM towards AW.

Blood supply pattern was analysed in all four types of SMT/APM arrangement, taking into account data obtained from multiple cross-sections derived from each specimen. The origin of APM arterial supply was traced back to either LCA or RCA—or, alternatively, both. Cross-sectional area of the arteries was estimated at the base of APM and compared among the individuals.

Histological examinations were conducted by means of classic staining methods (hematoxylin eosin, van Gieson’s stain with Goldner’s modification) under a stereo-microscope (Opta-Tech MN 800 Series, Warsaw, Poland) with Moticam 2000 camera Moticam 2000 2.0M (Motic Incorporation Ltd. Hong Kong) and Leica MZ8/MPS60 (Leica Microsystems Ltd Switzerland) photographic adapter with the use of a computer program Motic Images Plus 2.0. (Version 2.0 2006 Motic China Group CO., LTD.)

### Statistical analysis

Statistical analysis was performed using Wilcoxon's test and U-Mann-Whitney's test for dependent and independent variables, respectively. The statistical data is presented only for two morphological types: III and IV, as the low number of type I and II specimen did not allow for statistical analysis. The software used to perform analyses was R 2.15.1 (R Foundation for Statistical Computing, Vienna, Austria) and GraphPad 5 (GraphPad Software, Inc. La Jolla California, USA). P values <0.05 were considered statistically significant. Data is shown as median and interquartile range.

## Results

**Fig 2:**
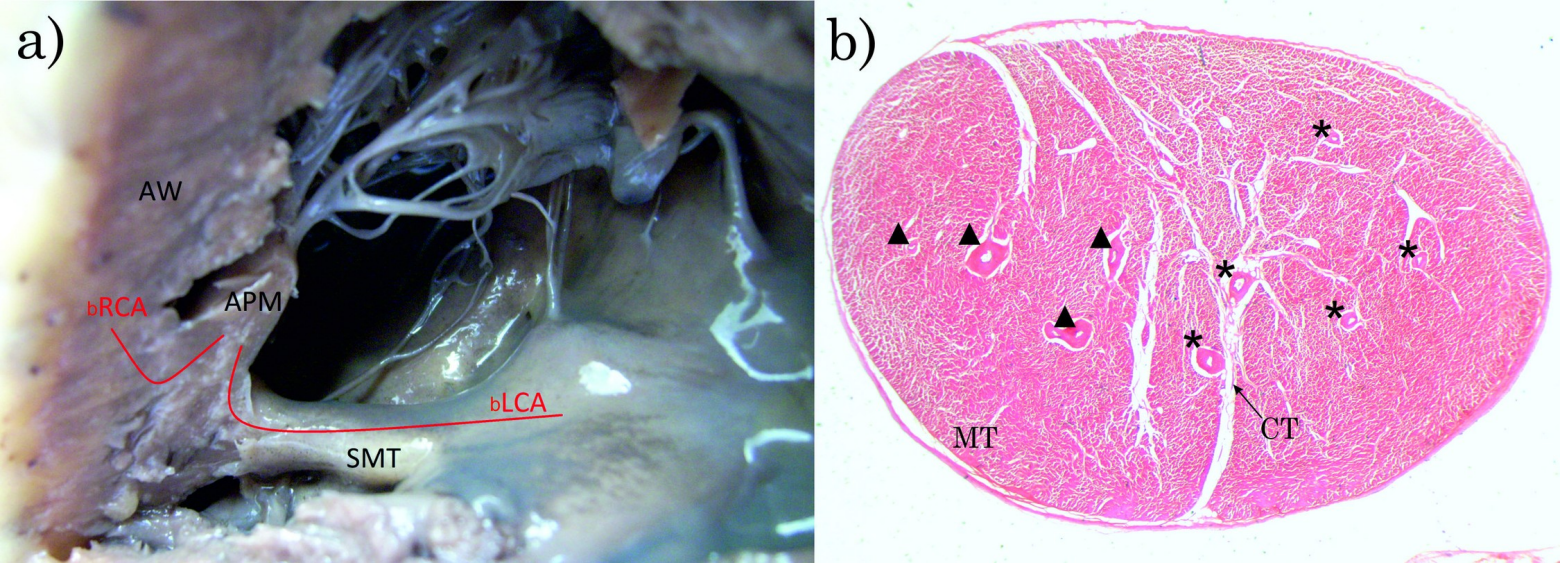
**Variety of vascularization of APM from two sources (LCA and RCA); a) View of the right ventricle of the heart; b) The cross-section of APM. a**) APM–anterior papillary muscle, bLCA–branches originated from the left coronary artery, bRCA–branches originated from the right coronary artery, SMT–septomarginal trabecula, AW–anterior wall of the right ventricle of the heart, **b**) *–branches originating from the left coronary artery, ▲–branches originating from the right coronary artery, MT–muscle tissue, CT–connective tissue. Haematoxylin and eosin stain. Magnification x20.

**Fig 3:**
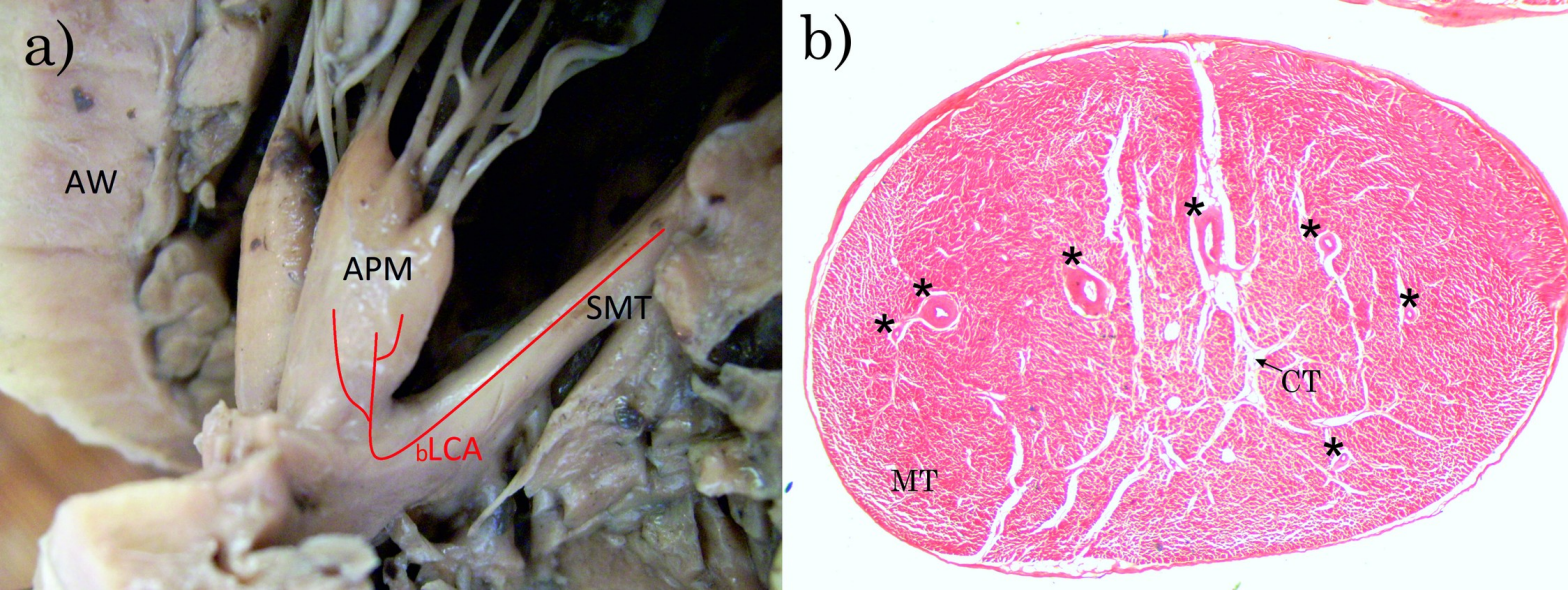
**Variety of vascularization of APM from one sources (LCA); a) View of the right ventricle of the heart; b) The cross-section of APM. a**) APM–anterior papillary muscle, bLCA–branches originating from the left coronary artery, SMT–septomarginal trabecula, AW–anterior wall of the right ventricle of the heart, **b**) *–branches originated from the left coronary artery, MT–muscle tissue, CT–connective tissue. Haematoxylin and eosin stain. Magnification x20.

In 28 hearts, out of 36, the blood supply of APM originated from both LCA and RCA branches (Fig 2A and 2B). Only in eight cases was the blood supplied only from one coronary artery—LCA (Fig 3A and 3B). RCA was never found to be the sole source of APM blood supply.

According to literature data, the number of arterial vessels may increase with age [17] or in case of myocardial ischemia [18], however phenomena of sexual dimorphism or age-related variability in the number of arteries have not been observed.

**Fig 4:**
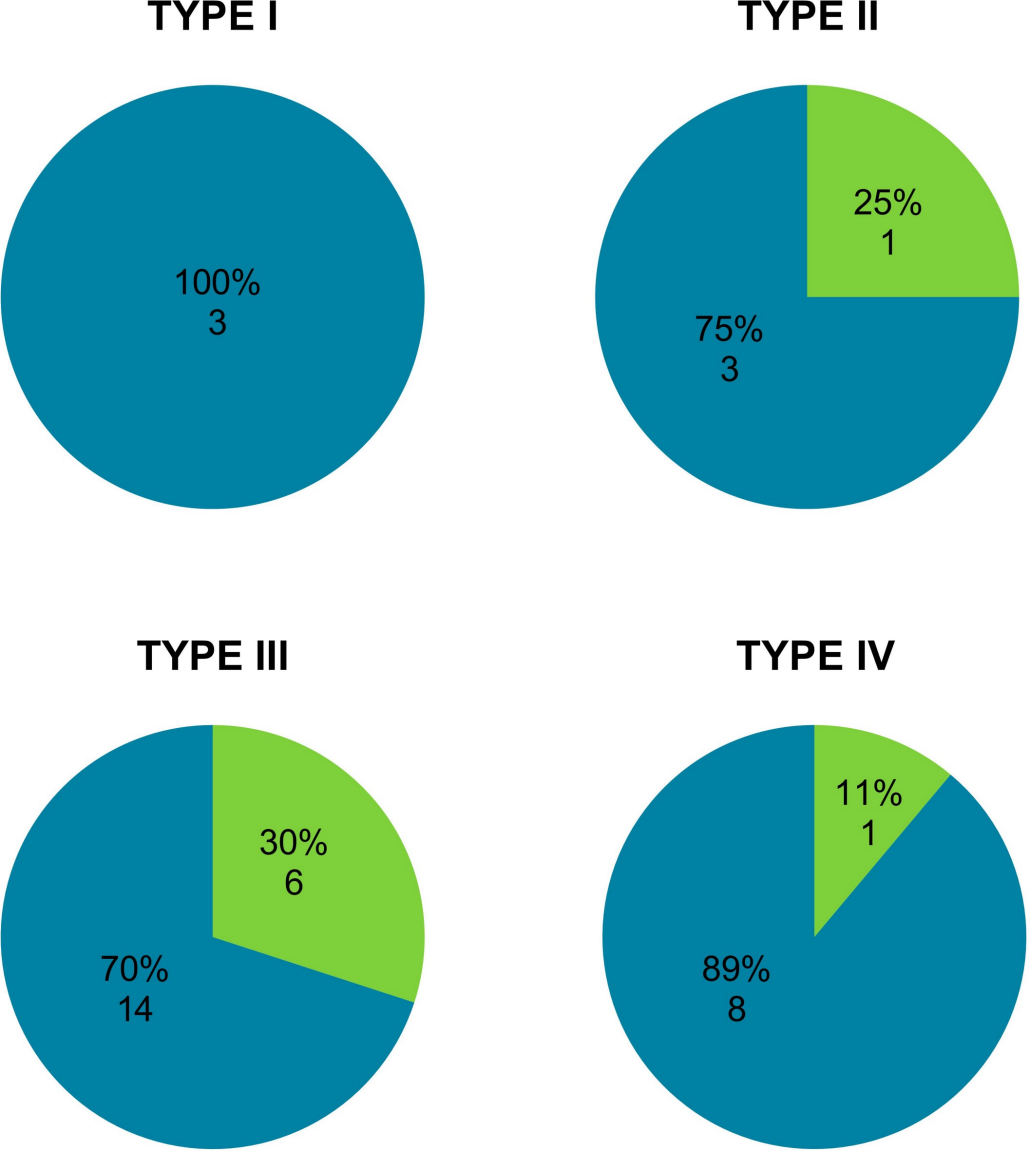
Proportional and quantitative comparison of variants of vascularization of APM by either one or two coronary arteries with the division into four types of connection of APM with SMT. Color code: Cerulean blue—variety of vascularization of APM from two sources (LCA and RCA). Green—variety of vascularization of APM from one sources (LCA). APM–anterior papillary muscle, LCA–left coronary artery, RCA–right left coronary artery, SMT–septomarginal trabecula.

Only three cases were qualified as type I. All had blood supply from two sources, both from LCA and RCA. In type II three cases had this type of vascular supply, moreover APM had a single source of blood supply, that is LCR, in one heart. In the most numerous type III, 70% (14) of APMs had vascular supply from both sources and the remaining 30% (6) from LCA alone. In type IV, which was next in frequency, 89% (8) cases had double blood supply of the examined muscle. One APM (11%) was supplied only by the branches of LCA (Fig 4).

**Fig 5:**
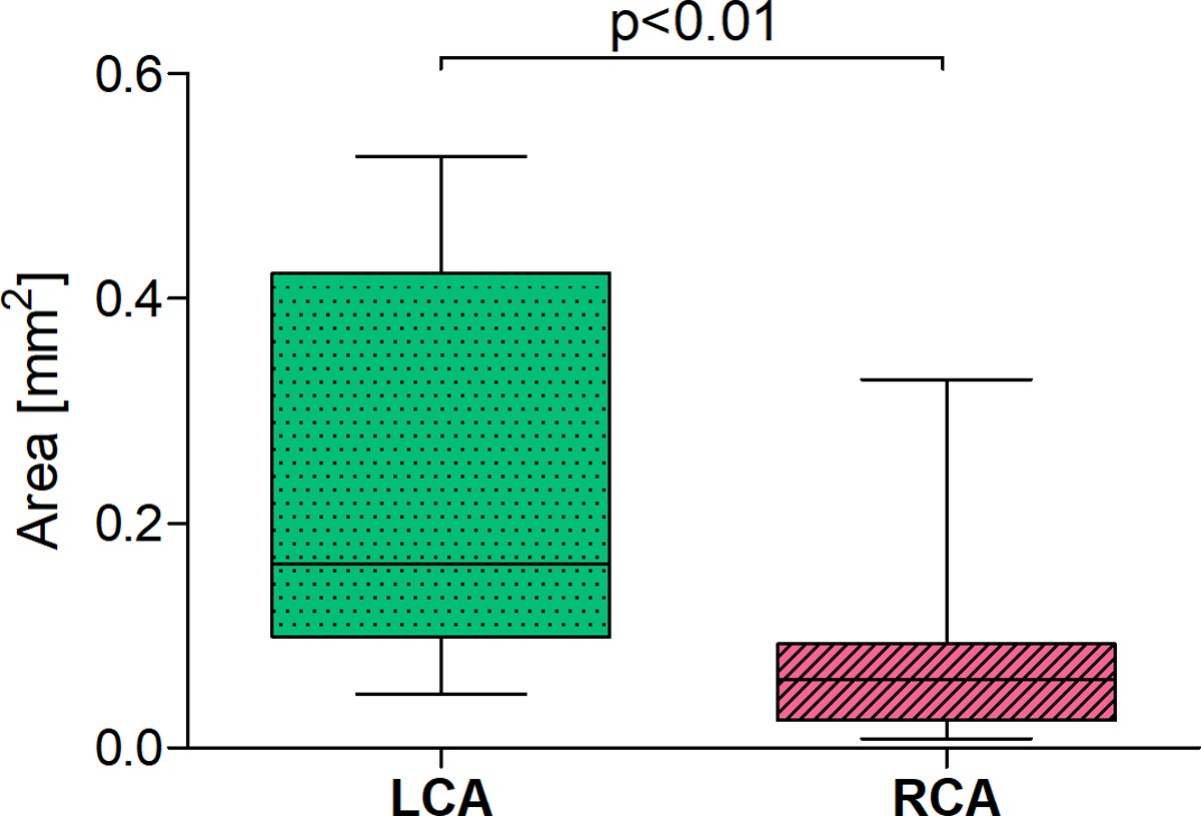
The values of total cross-section area of arteries originated from LCA and RCA, which supply APM; configuration type III: Permanent fusion of SMT within the APM. Date are shown as median and interquartile range. APM–anterior papillary muscle, LCA–left coronary artery, RCA–right left coronary artery, SMT–septomarginal trabecula.

The presented results indicate, that in case of double AMP blood supply in type III, an average of the total cross-section area of the arteries branching from LCA is statistically significantly bigger (over two and half times) compared with the arteries originating from RCA (Table 1 and Fig 5).

**Table 1:** The values of statistical characteristic of vascularization of APM originated from LCA and RCA; configuration type III: permanent fusion of SMT within the APM.

Coranary artery	N	X	SD	Min	Mediana	Max
Left	14	0.24	0.17	0.05	0.16	0.53
Right	14	0.09	0.10	0.01	0.06	0.33

LCA–left coronary artery, RCA–right left coronary artery, N–number of hearts, X–the average values of the areas of cross-section of arteries originated from LCA or RCA, APM–anterior papillary muscle, SMT–septomarginal trabecula, SD–standard deviation.

**Fig 6:**
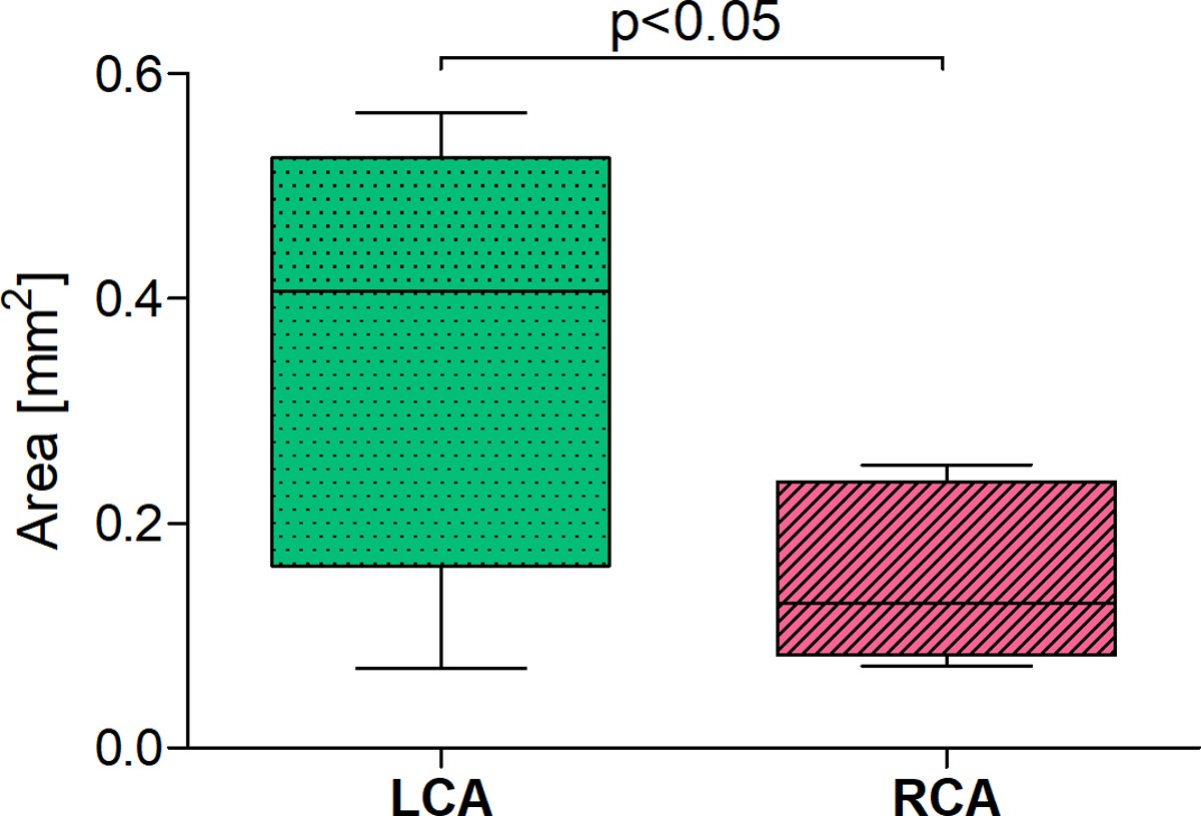
The values of total cross-section area of arteries originated from LCA and RCA, which supply APM; configuration type IV: SMT is structurally divided in two by APM. Date are shown as median and interquartile range. APM–anterior papillary muscle, LCA–left coronary artery, RCA–right left coronary artery, SMT–septomarginal trabecula.

The same applies to type IV. An average of the total cross-section area of the arteries branching from LCA is statistically significantly bigger (over two times) compared with the arteries originating from RCA (Table 2 and Fig 6).

**Table 2:** The values of statistical characteristic of vascularization of APM originated from LCA and RCA; configuration type IV: SMT is structurally divided in two by APM.

Coranary artery	N	X	SD	Min	Mediana	Max
Left	8	0.35	0.19	0.07	0.41	0.56
Right	8	0.15	0.08	0.07	0.13	0.25

LCA–left coronary artery, RCA–right left coronary artery, N–number of hearts, X–the average values of the areas of cross-section of arteries originated from LCA or RCA, APM–anterior papillary muscle, SMT–septomarginal trabecula, SD–standard deviation.

## Discussion

For a century now researchers have tried to work out the pattern of blood supply in the right and left ventricle of the human heart. Gross [19] and Gross and Kugel [20] suggested that the relative vascularization of RV was decreasing with age, as compared with the LV. Further studies, performed on the hearts of adults and children, did not confirm this observation [21]. It has been demonstrated, that in the healthy hearts of adults as well as children, a significant part of RV blood supply is provided by LCA. It has also been reported, that most of the arterial supply of APM is provided by LCA via the artery of SMT. It has been demonstrated, that arteries supplying APM can originate from LCA as well as from RCA [21–24].

In 1921, for the first time, Gross described APM vascular system and pointed out that it seemed to have stemmed from intraventricular branches (*rami interventriculares*) of LCA and from the anterior branches (*rami anteriores*) of RCA [19]. Moreover, he noted, that an artery originating from one of the first anterior septal arteries (*ramus limbi dextri*) could form anastomoses with the first posterior septal artery. On the other hand, Dobyns established, that apart from blood supply from arteries of SMT, meaning those originating from LCA, the muscle in question was also vascularised by RCA [22]. However, Dobyns' studies were performed on animal hearts, namely bovine, canine, porcine and chimpanzee. Dobyns cited contemporary Walmsley's anatomy textbook, which described SMT blood supply coming from main branches of LCA but not RCA. In agreement with Dobyns’ results, Farrer-Brown suggested that APM has double arterial blood supply and described the arteries penetrating this muscle as straight type [21]. Whilst analysing the vasculature of the RCA basin he observed that these were most often extensions of the right marginal branch of RCA, in some cases the arteries descended either from the right anterior ventricular branches or from both these arteries. Blood vessels derived from LCA had their source in the first anterior septal arteries [24].

Lüdinghausen et al, reported that APM receives blood via moderator band artery (*artery of the septomarginal trabelulae*) which descends from inferior branch of the anterior descending septal artery (ADSA) [25]. This publication pointed out that the most significant vessel was a right superior septal artery, which in some cases provided for both, SMT and APM. Moreover, arterial supply of the SMT itself did not always originated from ADSA. The report described six cases in which two-three dissectible arteries supplying SMT, derived from fourth or fifth branch of the anterior descending septal artery and the right superior septal artery. Most likely they reached as far as APM. A predominance of LCA in blood supply of the intraventricular septum, SMT and APM was also described. RCA ramifications were the sole blood supply of APM and SMT, and reached as far as the intraventricular septum in 3% of cases [26].

Angiographic studies by Topaz showed, in accordance with Reig's conclusions, that the first anterior septal artery was main the source of arteries supplying SMT–and extending towards APM [27].

The literature does not provide studies addressing the question of the exact participation of RCA and LCA in APM blood supply. Therefore we chose this question for the rationale behind our study to investigate the matter in detail using available classification of APM connection with SMT, including the forementioned four morphological types. We have established that, depending on the morphological type, SMT artery descending from LCA and its branches–are key for APM arterial supply and in some cases they form the sole blood supply of this muscle.

In our material as much as 78% of all APMs had blood supply originating from both LCA and RCA. Only in 22% cases APM blood supply was exclusively provided by one coronary artery, and that was always LCA. Never did we find APM arterial supply provided exclusively by RCA. In type III, in cases of double blood supply, average LCA-derived artery cross-section area, was significantly bigger than of those descending from RCA. It could be explained by the close APM/SMT connection, where muscle bundles run only towards APM. It seems that a distance between APM and AW may be the cause of poorer blood availability for APM originating from the RCA basin, resulting two times smaller cross-section area of RCA blood vessels as compared with LCA in type IV. It is also worth noting, that area of the LCA-derived arteries was 1,5 times bigger in type III, than in type IV, which can indicate a stronger relation with SMT in morphological type III.

Double blood supply of APM is inevitably related to possible connections between the LCA and RCA systems. There are several studies reporting formation of the anastomoses between these arteries within SMT and free wall of RV, but the authors do not mention the arterial supply of APM despite the anatomical proximity of this muscle [23,24,26,28]. In case of the collateral blood flow in the area adjoining APM, it seems well protected from ischemia, which points out at its role in preventing this process.

Analysis of the course of arteries within APMs showed the arteries branched forming peripheral rami towards the perimeter of the muscle. A similar pattern of arteries within APM was described by Farrer-Brown [21]. The group also compared this pattern of vascularisation with his earlier observations of blood supply of the LV papillary muscles [29].

The blood supply of the papillary muscles has a very important clinical aspect. Ischemia of papillary muscles can result in clinical dysfunction and a valvular prolapse syndrome [30]. In severe cases ischemia can lead to rupture of the papillary muscle, which without a surgical intervention may be lethal [9,11,31,32].

Researchers report that in no patient they examined following inferior myocardial infarction, with a double source of APM vascularization (0 of 6) there was mitral regurgitation observed. In contrast, 60 percent of the subjects (6 of 10) with a single vascularization source of APM suffered from mild or moderate regurgitation [15]. It can thus be concluded that double source of blood supply may play a protective role for AMP.

Our research confirms earlier suggestions of possible double blood supply of APM, but not necessarily in all individuals. The double blood supply of APM is most often observed in morphological types of APM/SMT arrangements, where the body of papillary muscle attached to the AW is also associated with the wall end of SMT. Most often, double APM vasculature can be observed in those morphological APM/SMT connections, in which the core of the muscle attached to the arterial wall is also precisely attached to the SMT wall. Therefore, addressing the initial hypothesis, we suggest that regardless of the connection type, LCA seems to be the main source of APM arterial vasculature.

## Supporting information

S1 Appendix Database of values of total cross-section area of arteries originated from LCA and RCA, or only from LCA which supply APM in four types. (XLSX)
